# Genome Sequence of a Klebsiella pneumoniae NDM-1 Producer Isolated in Quebec City

**DOI:** 10.1128/MRA.00829-19

**Published:** 2020-01-16

**Authors:** Maxime Déraspe, Jean Longtin, Paul H. Roy

**Affiliations:** a Centre de recherche en Infectiologie, Université Laval, Québec, QC, Canada; b Laboratoire de Santé Publique du Québec, Institut National de Santé Publique du Québec, Québec, QC, Canada; University of Arizona

## Abstract

We report here the complete genome sequence of Klebsiella pneumoniae CCRI-22199, isolated from a patient from India treated in Quebec City, Canada. Genes encoding beta-lactamases NDM-1 and CTX-M-15 were identified on two distinct plasmids. While the chromosome is similar to that of strain BAA-2146, CCRI-22199 provides a further example of rearrangements in plasmids.

## ANNOUNCEMENT

A multiresistant strain of Klebsiella pneumoniae, CCRI-22199, was an anal isolate from a 2-month-old infant who was a native of India. The strain was tested using Mueller-Hinton broth and agar and Etest, and it was resistant to third-generation cephalosporins, carbapenems, aminoglycosides, and ciprofloxacin but sensitive to trimethoprim-sulfamethoxazole, chloramphenicol, colistin, and tigecycline according to CLSI standards (1).

The genome was sequenced with DNA from a single-colony isolate, plated directly from a frozen stock from the clinical microbiology laboratory (maximum of 3 passages from initial isolation to DNA preparation). DNA was extracted using a KingFisher/Qiagen blood kit and prepared for Illumina MiSeq sequencing using a Nextera XT kit. DNA was also prepared according to the PacBio Template Preparation and Sequencing Guide (Pacific Biosciences, Menlo Park, CA) and sequenced by the single-molecule real-time (SMRT) technique using an RS II instrument (Pacific Biosciences) at the McGill University and Genome Quebec Innovation Centre. Illumina sequencing yielded 170,608 250-bp paired reads (341,216 in total), and PacBio sequencing yielded 140,824 reads with an average length of 7,806 nucleotides (nt). The genome was assembled *de novo* using the Hierarchical Genome Assembly Process (HGAP) (2). Further editing and manual annotation were carried out using Artemis (release 17.0.1) (3) with K. pneumoniae BAA-2146 (4) as a reference genome. Single-nucleotide polymorphisms (SNPs) in potential pseudogenes resulting from homopolymer tracts were resolved using NCBI BLAST (5) comparison between the PacBio and Illumina assemblies and were corrected in favor of Illumina results. Terminal overlaps were manually aligned and trimmed to yield circular chromosomal and plasmid sequences. All software used current versions and default parameters except for NCBI BLAST, where the low-complexity regions filter was turned off. Genome analysis revealed a chromosome with a length of 5,299,449 bp (57.5% G+C content) and plasmids with lengths of 185,957 bp (pKp199-1; 51.0% G+C content), 108,500 bp (pKp199-2; 55.3% G+C content), 48,698 bp (pKp199-3; 53.4% G+C content), and 3,359 bp (pKp199-4; 51.7% G+C content).

The chromosome was very similar to that of the well-characterized strain K. pneumoniae strain BAA-2146 (4), with 99.85% average nucleotide identity. Average nucleotide identity (ANI) was calculated using the pyani package and the Mummer average nucleotide alignment (ANIm) method (6). CCRI-22199 is sequence type 11 (ST11), and it shares 7 of the 11 genomic islands of BAA-2146. It has 11 copies of the group II intron Se.ma.I1, compared to 6 in BAA-2146.

**FIG 1:**
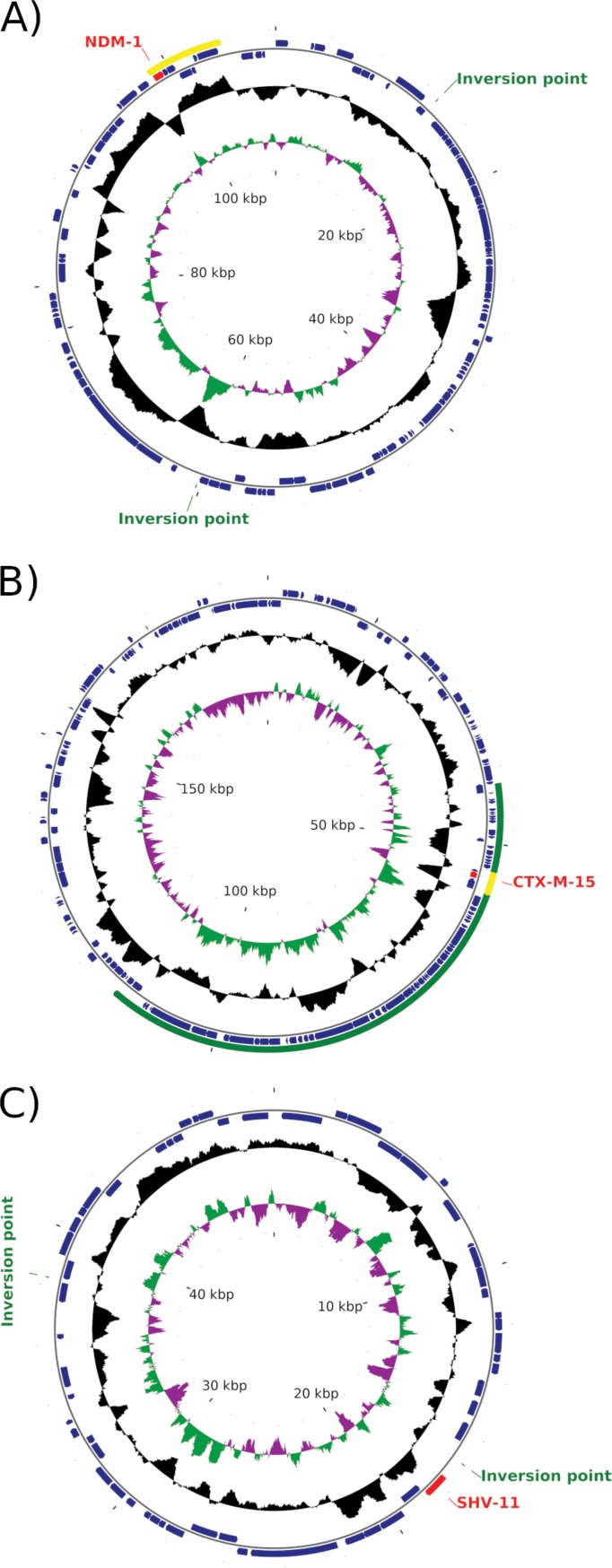
Map of plasmids pKp199-2 (A), pKp199-1 (B), and pKp199-3 (C) from K. pneumoniae CCRI-22199. The scales are indicated in the center. The first circle indicates G+C skew in green (positive [+]) and purple (negative −), and the second circle shows G+C content (deviation from the average) in black (+, outward, and −, inward). The third circle illustrates positions of coding sequences (CDSs) in minus (inward) and plus (outward) strands in dark blue. The red arcs show *bla* genes. The yellow arc in panel A shows the limited region of homology of pKp199-2 with pNDM-US from BAA-2146. The green arc in panel B represents the region of pKp199-1 not found in pKpn2146c (pCuAs) of BAA-2146; the yellow arc shows the limited region of homology with pKpn2146b (pHg) around the *bla*_CTX-M-15_ gene. “Inversion point” in panels A and C indicates the positions involved in rearrangements relative to the homologous plasmids pKOX_NDM-1 and pKpn2146b, respectively.

Plasmids pKp199-1, pKp199-2, and pKp199-3 encoded beta-lactamases CTX-M-15, NDM-1, and SHV-11, respectively. NCBI BLAST analysis showed that the NDM-1-encoding plasmid, pKp199-2 (Fig. 1A), was very similar to IncF plasmid pKOX_NDM-1 of Klebsiella oxytoca E718, which was isolated in Taiwan in 2010 (7). It contains an additional copy of IS*26* inserted into the *traG* gene, inactivating it and simultaneously inverting a 57-kb region of the plasmid (8). Similarity to the BAA-2146 IncA/C plasmid pNDM-US was limited to a 7-kb region around the *bla*_NDM-1_ gene.

Plasmid pKp199-1 (Fig. 1B) has both IncFIB and IncFII replication regions, as does pKp199-2, making it likely that a different replication region is used in each plasmid. The backbone of pKp199-1 is similar to that of pKpn2146c (pCuAs) with its arsenic and copper resistance operons and macrolide and tetracycline resistance genes. Only a 5-kb region of pKpn2146c is not found in pKp199-1, whereas a 72-kb region of pKp199-1 is absent in pKpn2146c. This region contains conjugal transfer genes, a type I restriction-modification system, a UvrD-type DNA helicase, and a gene encoding a methyl-accepting chemotaxis protein. Within the conjugal transfer region, and interrupting the *traK* gene, is a 3-kb mobile element homologous to a region of pKpn2146b (pHg) and encoding an IS*Ecp1* transposase and the CTX-M-15 extended-spectrum beta-lactamase.

Plasmid pKp199-3 (Fig. 1C) contains about 60% of plasmid pKpn2146b (pHg). The IncFIA and IncR regions are conserved, as are the mercury resistance operon, the phage shock protein region, a *qnr* quinolone resistance gene, a *bla*_SHV-11_ gene, and the *aac(6′)-Ib* and *aac(3)-IIe* aminoglycoside resistance genes. The *dfrA14* gene of pKpn2146b was absent in pKp199-3, correlating with the phenotypic sensitivity of CCRI-22199 to trimethoprim. Finally, plasmid pKp199-4 is a small (3.4-kb) cryptic plasmid.

Our work illustrates the rearrangement of plasmids compared to those in BAA-2146 (4). The *bla*_NDM-1_ gene-carrying plasmid has a completely different origin than that of BAA-2146, and it appears to be descended from pKOX_NDM-1 (7) by an IS*26*-mediated rearrangement (8). Plasmid pKp199-1 is larger than pKpn2146c; pKpn2146c lacks transfer genes. However, pKp199-1 appears to have acquired the *bla*_CTX-M-15_ gene by IS*Ecp1*-mediated transposition. Conversely, pKp199-3 may be derived from pKpn2146b by the loss of at least 4 distinct regions of pKpn2146b, including the *bla*_CTX-M-15_, *bla*_TEM-1_, and *bla*_OXA-1_ genes.

In summary, this work further underlines the roles of plasmid rearrangements and of mobile elements in the dissemination of carbapenemase and extended-spectrum beta-lactamase genes in Gram-negative bacteria.

### Data availability.

The chromosome and plasmid sequences were deposited in GenBank under accession numbers CP035540 (chromosome) and CP035536, CP035537, CP035538, and CP035539 (plasmids pKp199-1, pKp199-2, pKp1991-3, and pKp199-4, respectively). The PacBio and Illumina reads were deposited in the Sequence Read Archive under accession numbers SRX6452124 and SRX7007143, respectively.
